# Risk Factors and Treatment for Hemorrhage after Pancreaticoduodenectomy: A Case Series of 423 Patients

**DOI:** 10.1155/2016/2815693

**Published:** 2016-11-16

**Authors:** Feng Gao, Jianguo Li, Shengwei Quan, Fujun Li, Donglai Ma, Lei Yao, Ping Zhang

**Affiliations:** ^1^Department of General Surgery, Second Affiliated Hospital of Harbin Medical University, Harbin 150000, China; ^2^Department of General Surgery, First Affiliated Hospital of Jilin University, Changchun 130000, China; ^3^Department of General Surgery, Heilongjiang Province Hospital, Harbin 150000, China; ^4^Department of General Surgery, Heilongjiang Province Land Reclamation Headquarters General Hospital, Harbin 150000, China

## Abstract

The study aimed to investigate the risk factors of postpancreatectomy hemorrhage (PPH) after pancreaticoduodenectomy (PD). A retrospective analysis of 423 patients who underwent PD between January 2008 and January 2014 was conducted. The overall incidence and all-cause mortality of PPH were 9.9% (42/423) and 2.1% (9/423), respectively. Independent risk factors of early PPH were revascularization (odds ratio (OR) = 6.786; 95% confidence interval (95% CI): 1.785–25.792; *P* = 0.005), history of abdominal surgery (OR = 5.009; 95% CI: 1.968–12.749; *P* = 0.001), and preoperative albumin levels (OR = 4.863; 95% CI: 1.962–12.005; *P* = 0.001). Independent risk factors of late PPH included postoperative pancreatic leakage (OR = 4.696; 95% CI: 1.605–13.740; *P* = 0.005), postoperative biliary fistula (OR = 6.096; 95% CI: 1.575–23.598; *P* = 0.009), postoperative abdominal infection (OR = 4.605; 95% CI: 1.108–19.144; *P* = 0.036), revascularization (OR = 9.943; 95% CI: 1.900–52.042; *P* = 0.007), history of abdominal surgery (OR = 8.790; 95% CI: 2.779–27.806; *P* < 0.001), and preoperative albumin levels (OR = 5.563; 95% CI: 1.845–16.776; *P* = 0.002).

## 1. Introduction

Pancreaticoduodenectomy (PD) is a complex procedure involving extensive resection with significant risk of postoperative complication such as pancreatic and biliary fistula, delayed gastric emptying, abscess formation, and postpancreatectomy hemorrhage (PPH) [1]. Despite improvements in surgical approaches, the rate of complications remains high at 30–50% and the mortality is still significant, even in high volume centers [1–4].

Due to vast improvements in care in the last decades, the incidence of PPH is relatively low at 3–10% but nevertheless has a high mortality rate at 20–50% [1, 5–17]. Therefore, when PPH occurs, it has to be considered a life-threatening condition. In addition, there is no consensus or guideline about the management of PPH, and the reported clinical experience is limited [18, 19]. In 2005 and 2007, the International Study Group on Pancreatic Fistula (ISPGF) proposed a consensus about pancreatic fistulae [20] and PPH [17]. It emphasizes that a clear definition of PPH and its risk factors is still lacking [17].

PPH may occur either early or late. Early PPH is usually iatrogenic and results from arterial injury or suboptimal hemostasis [7, 8, 21, 22]. Early PPH usually requires emergency reoperation. On the other hand, late PPH is usually a complication of pancreatic fistula or abdominal abscess [10, 13, 23] and is assessed and treated using angiography and endoscopy. Nevertheless, the prognosis of PPH remains poor despite advances in imaging and treatment, and no preventive measure is recognized [1].

Therefore, identifying patients who are at higher risk of PPH is clinically relevant. The aim of this study was to review retrospectively the clinical data of 423 patients who underwent PD at a single center and to identify risk factors of PPH.

## 2. Materials and Methods

### 2.1. Patients

Between January 2008 and January 2014, 423 patients underwent PD at the Second Affiliated Hospital of Harbin Medical Science University. Inclusion criteria were (1) diagnoses of ampullary cancer, distal cholangiocarcinoma, duodenal adenocarcinoma, pancreatic cancer, partial benign pancreaticoduodenal tumor, chronic pancreatitis, and/or invasion of other tumors into the head of the pancreas and duodenum, as confirmed prior to surgery; (2) PD performed and diagnoses confirmed by pathological examination; (3) <75 years old; (4) normal cardiopulmonary functions and coagulation tests; and (5) adequate liver function: total bilirubin <200 *μ*mol/L and albumin >30 g/L. Exclusion criteria were (1) distant metastasis confirmed by imaging or (2) loss to follow-up. Indications for PD were (1) confirmed diagnosis of pancreatic lesions for which PD was the first treatment choice; (2) early detection and good physical status without distant metastasis; (3) age <75 years old; (4) normal cardiopulmonary functions and coagulation; (5) adequate liver function prior to surgery: total bilirubin <200 *μ*mol/L and albumin >30 g/L; and (6) no tumor invasion of a surrounding major blood vessels. For patients with low albumin levels before surgery, measures were taken to increase albumin levels, mainly using albumin supplementation and nutritional support. Only patients with albumin >30 g/L underwent surgery. Patients who had low albumin levels but underwent surgery because of corrected levels were classified as having low albumin levels for the analyses.

This study was approved by the ethics committee of the Harbin Medical Science University. The need for individual consent was waived by the committee because of the retrospective nature of the study.

### 2.2. Surgery and Perioperative Management

Routine blood and coagulation tests were monitored preoperatively, and patients with elevated amino transferase or bilirubin received liver protection therapy using 250 mL of 5% glucose solution with 120 mg of glycyrrhizin, once per day, intravenously. Patients with serum bilirubin >171 *μ*mol/L (i.e., those with a suspicion of biliary function impairment and probably with abnormal absorption of vitamin K) received intramuscular injections of vitamin K. Patients with serum bilirubin >200 *μ*mol/L underwent percutaneous transhepatic biliary drainage to prevent jaundice.

All patients underwent standard PD with reconstruction using the Child's approach. The approach to managing the pancreatic duct was determined according to the pancreatic texture and dilation of the pancreatic duct. Patients with pancreatic duct ≥3 mm and with a hard residual pancreas usually underwent mucosa-to-mucosa end-to-side pancreaticojejunostomy (*n* = 307). Patients with soft residual pancreas or normal pancreatic duct usually underwent end-to-end or end-to-side pancreaticojejunostomy, in which the pancreatic stump was invaginated 2-3 cm and sutured using two interrupted layers (*n* = 116). In all patients, a drainage tube was placed in the pancreatic duct, with the distal end inserted into the jejunum to drain the pancreatic fluids into the distal jejunum or externally via the distal jejunum lumen to avoid contact of pancreatic and intestinal fluids on the anastomosis to reduce the risk of pancreatic fistula.

After surgery, patients were routinely fasted and received gastrointestinal decompression for 5–10 days, antibiotics, proton pump inhibitors, somatostatin, and other therapies as required. Somatostatin was given for 3–7 days according to the pancreas texture.

### 2.3. Evaluation of PPH

When PPH occurred, the patient routinely underwent abdominal CT examination. For patients with gastrointestinal bleeding, endoscopy was performed to identify the bleeding site and to treat the source of the bleeding using electrocoagulation or clipping. If endoscopy failed, then the patient underwent surgery. Patients with early abdominal bleeding (mild to moderate) first received nonsurgical treatment. Patients with severe abdominal bleeding went directly to laparotomy. Patients with late abdominal hemorrhage first received vascular embolization.

Bleeding was divided into abdominal PPH and digestive duct PPH. According to the criteria issued by the International Study Group of Pancreatic Surgery (ISGPS) [17], PPH was divided into early (≤24 h) and late (>24 h) PPH. PPH was further divided into mild (grade A, no special intervention is required apart from symptomatic support) and severe PPH (grade B, intervention is required; or grade C, patient condition is critical) according to the severity [17]: (1) mild: hemoglobin reduction of ≤30 g/L within 24 h, with stable hemodynamics or maintainable circulation with adequate fluid replacement or (2) severe: hemoglobin reduction of >30 g/L within 24 h, with significant hypovolemic shock, unrecoverable circulation with adequate fluid replacement, necessity of infusion of >3 U of packed red blood cells, and requirement for intervention or surgery.

Pancreatic fistula was defined according to the ISGPS [20], based on the amylase content of the abdominal drainage fluid being >3 times serum levels 3 days after PD. Pancreatic fistula was divided into grade A, B, or C: (1) grade A: transient, absence of symptoms, or increased amylase in drainage tube with nonsignificant imaging changes, and disappearance of drainage fluid within 3 weeks, especially at the first postoperative week; (2) grade B: fever, vomiting, aphagosis, abdominal pain and distension, and peripancreatic fluid in imaging; or (3) grade C: critical condition, unstable vital signs, more apparent abdominal symptoms and vital signs compared to grade B, significant peripancreatic fluid on imaging, requirement for reoperation, likelihood of complication with sepsis, multiple organ dysfunction syndrome, respiratory failure, or death.

Biliary fistula was defined as continuous drainage with abundant bile for more than 5 days. Postoperative abdominal infection was defined as persistent fever, increased leukocytosis, drainage of pus from the peritoneal drainage tube, and ascites on B-mode ultrasound or CT [24]. Finally, postoperative mortality was defined as death after PD and during hospitalization.

### 2.4. Treatment of PPH

Treatment of PPH was divided into nonsurgical and surgical treatments. The selection of the appropriate strategy was made by the surgeon. Nonsurgical treatment was preferred for patients with grades A and B PPH and included fasting, acid suppression, blood transfusion, somatostatin, hemostatic drugs, and other appropriate measures. Surgery was performed in patients with grade C PPH and ineffective nonsurgical treatment and included exploratory laparotomy and hemostasis, total pancreatectomy, repair of pancreaticoenteric anastomosis, pancreaticobiliary diversion (PBD) (Roux-en-Y pancreatico-jejunum reanastomosis), pancreatic-gastric anastomosis, internal and external drainage of pancreatic duct, and abscess drainage. The surgical approach was based on localized injury caused by pancreatic leakage and the severity degree of abdominal infection. If the intraoperative assessment of abdominal infection caused by pancreatic leakage was severe, the risk of a second pancreaticojejunostomy was very high and anastomosis could not be performed again. Then, total pancreatectomy was selected.

Patients suffering from PPH after PD were followed up more intensively every four weeks for 3 months. The follow-up interval was one month. Follow-up was performed via telephone and/or visits to the outpatient clinic. Follow-up parameters included severe postsurgical complications (bile leakage, pancreatic leakage, abdominal infection, etc.), tumor recurrence, and metastases. In the presence of any complain from the patient or the family, the patient was seen at the outpatient clinic.

### 2.5. Data Collection

Risk factors of PPH were divided into three categories: (1) preoperative factors: age, gender, body mass index (BMI), hypertension, diabetes, coronary disease, smoking, alcohol, history of abdominal surgery, serum bilirubin, preoperative albumin level, and drainage; (2) intraoperative factors: surgical approach, intraoperative transfusion, use of anastomosis, revascularization, pancreatic duct diameter, and anastomotic approach; and (3) postoperative factors: pancreatic leakage, biliary fistula, abdominal infection, and use of somatostatin.

Total bilirubin was mainly used to detect the presence of liver disease or biliary tract abnormality. Normal total bilirubin levels range from 3.4 to 17.1 *μ*mol/L; 17.2–34.2 *μ*mol/L was considered as recessive jaundice; 34.3–170 *μ*mol/L was considered as mild jaundice; 171–340 *μ*mol/L was considered as moderate jaundice; and >340 *μ*mol/L was considered as severe jaundice.

### 2.6. Statistical Analysis

Statistical analyses were performed using SPSS 19.0 (IBM, Armonk, NY, USA). Continuous data are presented as means ± standard deviation and were analyzed using the Student* t*-test. Categorical data are presented as frequencies and were analyzed using the chi-square test or the Fisher exact test, as appropriate. Variables with significant associations in univariate analyses were included in multivariate analyses. Independent risk factors were identified using the logistic regression analysis (enter method). Associations are presented as odds ratios (OR) and 95% confidence intervals (95% CI). Two-sided *P* values < 0.05 were considered statistically significant.

## 3. Results

### 3.1. Characteristics of the Patients

There were 247 men and 176 women, aged 57.3 ± 10.2 (range 22–74) years old. Malignant tumors or stromal tumors with malignant potential accounted for 88.4% (374/423) of the cases and benign diseases accounted for 11.6% (49/423). Postoperative pathological examinations confirmed 152 periampullary tumors (ampullary carcinoma, distal bile duct cancer, and duodenal papillary carcinoma of lower segment), 86 pancreatic ductal adenocarcinomas, 73 bile duct carcinomas, 31 duodenal adenocarcinomas, seven pancreatic neuroendocrine tumors, nine cystadenocarcinomas of the pancreas, seven malignant stromal tumors, six intraductal papillary mucinous neoplasms, three invasions by a gastrointestinal cancer, 12 chronic pancreatitides, eight pancreatic cystadenomas, six pancreatic solid pseudopapillary neoplasms, three duodenal papillary adenomas, three islet cell adenomas, one ectopic pancreas, and 16 other tumors.

### 3.2. Complications

Of the 423 PD patients, the incidence of PPH was 9.9% (42/423). The postoperative incidence of pancreatic leakage, bile leakage, and abdominal infection was 23.2% (98/423), 5.9% (25/423), and 4.7% (20/423), respectively.

Among the 42 patients (33 male; 9 female) with PPH, 23 had early and 19 late PPH, 14 arising from the digestive tract and 28 from elsewhere in the abdomen. There were 4 grade A, 28 grade B, and 10 grade C PPH. Most of the late abdominal PPH events were arterial (57.9%, 11/19).

In this study, 19 patients suffered from late PPH and eight patients died (five after surgical treatment and three after nonsurgical treatment). All the 19 patients underwent angiography examination or gastrointestinal endoscopy at first. Among the 11 surviving patients, nine had abdominal bleeding. Among these nine patients, the bleeding site was detected by DSA in seven, and they all underwent hemostasis with embolization treatment. The exact bleeding site could not be identified in two patients. There were two cases of gastrointestinal tract bleeding. One underwent endoscopy and achieved hemostasis. The bleeding site in the other case could not be identified, but the bleeding resolved spontaneously.

Among the eight mortality cases, there were three patients who suffered from bleeding after embolization. Two patients whose bleeding sites were detected as the portal vein underwent surgery. Two other cases that underwent angiography and embolization died from organ failure. One patient diagnosed with gastrointestinal tract bleeding underwent endoscopy, but he died from organ failure.

### 3.3. Univariate Analyses of Risk Factors of PPH

Univariate analysis of preoperative factors for the occurrence of PPH (Table 1) showed that a history of abdominal surgery and low albumin levels were was significantly associated with early PPH and late PPH.

Similar analysis of intraoperative and postoperative factors (Table 2) demonstrates that revascularization was associated with early leakage while pancreatic fistula biliary fistula, abdominal abscess, and revascularization were associated with late PPH.

### 3.4. Multivariate Analyses of Risk Factors of PPH

Multivariate analysis (Table 3) demonstrated that revascularization, past history of abdominal surgery, and preoperative albumin levels were independently associated with early PPH.

The independent risk factors for late PPH (Table 4) included postoperative pancreatic fistula postoperative bile fistula postoperative abdominal abscess revascularization, history of abdominal surgery, and preoperative albumin levels.

### 3.5. Mortality

The overall mortality from PPH (Table 5) was 2.1% (9/423). All patients with late PPH underwent DSA before surgery, while patients with early PPH did not undergo DSA.

## 4. Discussion

The incidence of PPH of 9.9% in this study is slightly higher than the 5.7–8.8% reported previously [1, 13]. A number of factors could be involved in this difference. In the early period of this study, there was no permanent team for pancreatic surgery, resulting in a low degree of specialization and a lack of multidisciplinary cooperation. In the last 2 years of the study period, the incidence of PPH decreased to 6.3%, suggesting that establishing a professional team dedicated to pancreatic surgery can significantly reduce iatrogenic bleeding and the occurrence of PPH. Our mortality rate for PPH of 21.4% is consistent with the 11–47% previously reported [1, 14].

This study showed that vascular reconstruction and history of previous abdominal surgery were independent risk factors of early PPH, which support the view proposed by the ISGPS that early surgery should be considered for early PPH [17]. This study also found that bleeding from residual uncinate process was another important cause of early PPH [25] and accounted for 8.7% (2/23) of the early PPH cases. The uncinate process is located deeply with a number of arterial branches within it; therefore, mass ligation is prone to incomplete hemostasis, perhaps contributing to early PPH. In addition, necrosis of the residual uncinate process may cause late PPH. Total resection of the uncinate process would represent a good method of preventing hemorrhage from any residual pancreatic tissue. The authors adopted the method of resecting the tissue to the right side of the superior mesenteric artery sheath, including the uncinate process, resulting in elimination of residual postoperative bleeding of the uncinate process. DSA examination and TAE can be used in patients with early PPH in which nonsurgical treatment is ineffective [26]. CTA could help the diagnosis of delayed arterial hemorrhage with a positive rate above 95% [27]. Meanwhile, TAE can be applied simultaneously if the bleeding parts can be accurately identified [28].

Postoperative pancreatic fistula and abdominal infection are independent risk factors of late PPH. Data from the ISGPS also revealed that pancreatic fistulae, abdominal infection, vascular corrosion by digestive juices, and other postoperative complications were the main reasons for late PPH. Late PPH is also known as pancreatic fistula-related bleeding. In the 19 cases with PPH in this study, 94.7% (18/19) also showed pancreatic fistula, and 26.5% (5/19) also had abdominal infection. Among the nine deceased patients, cases of late PPH were always accompanied by pancreatic leakage and this rate was higher than in previous studies [29]. It might be because some patients might suffer from missed or occult pancreatic fistula or collections. Early diagnosis of pancreatic fistula relies on close observation of the abdominal drain including color and amount of abdominal drainage fluid. Early determination of amylase after surgery is vital for early diagnosis in patients whose recovery is not smooth. Patients with poor drainage and symptoms of intra-abdominal abscess/collection should undergo ultrasound- or CT-guided puncture as early as possible to avoid the accumulation of pancreatic juice and abscesses and corrosion of vessels [30, 31].

In this study, most of the late abdominal PPH events were arterial (57.9%, 11/19). Normally, a small amount of bleeding will occur 6 h to 10 d before late PPH, which is called “sentinel bleeding” [32] and occurs in 45% of the patients with late PPH. This indicates that attention should be paid to the sentinel bleeding in predicting late PPH, and effective measure should then be taken to prevent PPH. It has been suggested that angiography should be immediately conducted in patients with sentinel bleeding in order to provide early treatments [33].

**Figure 1:**
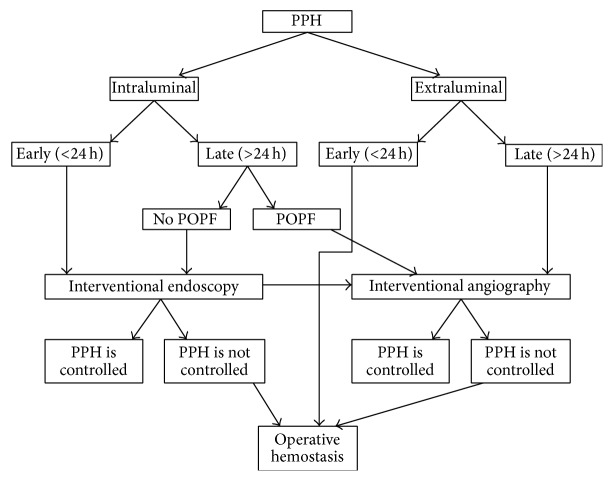
Suggested management algorithm of postpancreatectomy hemorrhage (PPH). POPF: postoperative pancreatic fistula.

Five patients treated with preoperative DSA or TAE treatment died after surgery because hemostasis could not be achieved. Late PPH is associated with more unstable hemodynamics. Bleeding is usually more important and treatment rarely achieves good results. Based on our experience, we summarized the clinical pathway for the treatment of PPH in Figure 1. The combined use of medical therapy (such as somatostatin), angiography, and laparoscopy based on this algorithm could have contributed to the relatively low mortality rate observed in the present study. Of course, the patients have to be carefully selected and the absence of metastasis has to have been proven before any surgery in order to avoid unnecessary surgery and risks. An Italian study of 544 hospitals showed that 9% of pancreatic resections were performed in patients with distant metastases [34], which should be avoided. Nevertheless, other techniques should be explored to further lower this mortality rate, such as endovascular techniques [35].

The present study found that the independent risk factors that correlated with early/late PPH included history of abdominal surgery and preoperative albumin level. Indeed, surgery may more difficult in patients who have a history of abdominal surgery because of the eventual presence of scars, fibrosis, adhesions, clips, and changed anatomy. Postoperative scar tissue is rich in blood vessels and it is easy to make them bleed during surgery and hemostasis is difficult because of the absence of major blood vessels. These factors could lead to a higher incidence of PPH.

An excessively low preoperative serum albumin levels indicate poor liver function and malnutrition and often indicate a poor tolerance to surgery and unfavorable anastomosis healing, prone to ascites and pancreatic fistula, which were also the main risk factors of PPH. This is supported by a previous study that showed that the nutritional risk index was associated with the risk of PPH [31].

This study suffers from some limitations. The sample size was limited, preventing the observation of some possible associations. In addition, it was from a single institution with changing practice over time. Additional multicenter studies should be performed to confirm these results.

## 5. Conclusions

In conclusion, results suggest that PPH could be prevented by improving the preoperative nutrition of patients, accurate PD, protection of blood vessels, proper hemostasis, and prevention and treatment of pancreatic leakage and abdominal infection.

## Figures and Tables

**Table 1:** Univariate analyses of preoperative factors for early and late PPH.

Variables	Early PPH (*n* = 23)	No PPH (*n* = 381)	*P*	Late PPH (*n* = 19)	No PPH (*n* = 381)	*P*
Preoperative serum bilirubin (*μ*mol/L)			0.071			0.394
>171	9	83		6	83	
≤171	14	298		13	298	
PBD			0.738			0.717
Yes	4	24		2	24	
No	7	53		7	53	
Previous abdominal surgery			0.001			<0.001
Yes	10	59		9	59	
No	13	322		10	322	
Worst preoperative albumin levels			0.001			0.002
Low (<30 g/L)	11	64		8	64	
Normal (>30 g/L)	12	317		11	317	

PPH: postpancreatectomy hemorrhage; PBD: pancreaticobiliary diversion.

**Table 2:** Univariate analyses of intraoperative and postoperative factors for early and late PPH.

Variables	Early PPH (*n* = 23)	No PPH (*n* = 381)	*P*	Late PPH (*n* = 19)	No PPH (*n* = 381)	*P*
Anastomosis type			0.223			0.181
Nonmucosa to mucosa	9	99		8	99	
Mucosa to mucosa	14	282		11	282	
Postoperative pancreatic fistula			>0.99			0.011
Yes	0	88		10	88	
No	0	293		9	293	
Preoperative biliary fistula			>0.99			0.024
Yes	0	21		4	21	
No	0	360		15	360	
Postoperative abdominal abscess			>0.99			0.011
Yes	0	16		4	16	
No	0	365		15	365	
Vascular reconstruction			0.018			0.047
Yes	4	15		3	15	
No	19	366		16	366	
Intraoperative pancreatic duct diameter			0.654			0.854
≥3 mm	10	135		12	254	
<3 mm	13	265		7	127	

PPH: postpancreatectomy hemorrhage; PPPD: pylorus-preserving pancreaticoduodenectomy; PD: pancreaticoduodenectomy.

**Table 3:** Multivariate analysis of risk factors of early PPH.

Variables	*P*	OR	95% CI	
			Lower	Upper
Vascular reconstruction	0.005	6.786	1.785	25.792
History of abdominal surgery	0.001	5.009	1.968	12.749
Preoperative albumin level	0.001	4.863	1.962	12.005

PPH: postpancreatectomy hemorrhage; OR: odds ratio; 95% CI: 95% confidence interval.

**Table 4:** Multivariate analysis of risk factors of late PPH.

Variables	*P*	OR	95% CI	
			Upper	Lower
Preoperative pancreatic leakage	0.005	4.696	1.605	13.740
Postoperative bile leakage	0.009	6.096	1.575	23.598
Postoperative abdominal infection	0.036	4.605	1.108	19.144
Vascular reconstruction	0.007	9.943	1.900	52.042
Past history of abdominal surgery	<0.001	8.790	2.779	27.806
Preoperative albumin level	0.002	5.563	1.845	16.776

PPH: postpancreatectomy hemorrhage; OR: odds ratio; 95% CI: 95% confidence interval.

**Table 5:** Nine postoperative deaths from PPH.

Case	Age/ gender	Primary disease	Pancreatic leakage	Bleeding time after surgery (h)	Bleeding type	Treatment approach	Source	Cause of death
1	38/M	Pancreatic ductal adenocarcinoma	No	7	Early intra-abdominal bleeding	Surgery	Section of residual uncinate process of pancreas	Rebleeding, MODS
2	42/M	Ampullary carcinoma	No	130	Late intra-abdominal bleeding	DSA, TAE, surgery	Mesenteric artery	Hepatic failure, renal failure
3	42/F	Cystadenocarcinoma of pancreas	Yes	118	Late intra-abdominal bleeding	DSA, TAE, surgery	Gastroduodenal artery	Rebleeding, MODS
4	54/M	Pancreatic ductal adenocarcinoma	Yes	152	Late intra-abdominal bleeding	DSA, surgery	Portal vein	Rebleeding, MODS
5	57/M	Distal bile duct carcinoma	Yes	126	Late intra-abdominal bleeding	DSA, TAE	Gastroduodenal artery	Hepatic failure
6	65/F	Distal bile duct carcinoma	Yes	134	Late intra-abdominal bleeding	DSA, TAE, surgery	Gastroduodenal artery	Hepatic failure, renal failure
7	68/M	Cystadenocarcinoma of pancreas	Yes	142	Late intra-abdominal bleeding	DSA, surgery	Portal vein	Rebleeding, MODS
8	71/M	Ampullary carcinoma	Yes	128	Late gastrointestinal bleeding	Gastroscopy, DSA, TAE	Pancreaticojejunostomy anastomosis	Abdominal infection; MODS
9	72/M	Ampullary carcinoma	Yes	136	Late gastrointestinal bleeding	Gastroscopy, DSA	Unknown bleeding part	MODS

MODS: multiple organ dysfunction syndrome; DSA: digital subtraction angiography; TAE: transarterial embolization; M: male; F: female.
